# Supplementary material for: Heat adaptation of phage T7 under an extended genetic code
Source: Virus Evol. 2021 Dec 1;7(2):veab100. doi: 10.1093/ve/veab100 (PMC8923235; doi:10.1093/ve/veab100)
Supplement: veab100_Supp [file veab100_supp.zip › Sequencing_Analysis.zip!/Sequencing_Analysis/S2/evidence/SNP_14.html]

BRESEQ :: Evidence


| Predicted mutation | | | | | | |
| --- | --- | --- | --- | --- | --- | --- |
| evidence | seq id | position | mutation | annotation | gene | description |
| RA | NC\_001604.1 | 35,095 | A→G | intergenic (–/–) | – / – | –/– |

| Read alignment evidence... | | | | | | | | | | | |
| --- | --- | --- | --- | --- | --- | --- | --- | --- | --- | --- | --- |
|  | seq id | position | | ref | new | freq | score (cons/poly) | reads | annotation | genes | product |
| \* | NC\_001604.1 | 35,095 | 0 | A | G | 99.5% | 14130.3 / NA | 4561 | intergenic (–/–) | *–/–* | –/– |
| Reads supporting (aligned to +/- strand):  **ref** base A (4/12);  **major** base G (2136/2383);  **minor** base T (10/14);  **total** (2151/2410) | | | | | | | | | | | |
| Fisher's exact test for biased strand distribution *p*-value = 6.84e-01 | | | | | | | | | | | |
| Kolmogorov-Smirnov test that lower quality scores support variant *p*-value = 1.00e+00 | | | | | | | | | | | |

Only 100 of 4561 total aligned reads displayed.  

`CTCTCTGACAAAGGCTTGGGGTCCAGCCGATGGCTACACGACCATCGAGTTACGTCGAGTAACCTCCACTACCGACCGATTGGTTGACTTTACGGATGGTTCAATCCTCCGCGCGTATGACCTTAACGTCGCTCAGATTCAAACGATGCACGTAGCGGAAGAGGCCCGTGACCTCACTACGGATACTATCGGTGTCAATAACGATGGTCACTTGGATGCTCGTGGTCGTCGAATTGTGAACCTAGCGAACGCCGTGGATGACCGCGATGCTGTTCCGTTTGGTCAACTAAAGACCATGAACCAGAACTCATGGCAAGCACGTAATGAAGCCTTACAGTTCCGTAATGAGGCTGAGACTTTCAGAAACCAAGCGGAGGGCTTTAAGAACGAGTCCAGTACCAACGCTACGAACACAAAGCAGTGGCGCGATGAGACCAAGGGTTTCCGAGACGAAGCCAAGCGGTTCAAGAATACGGCTGGTCAATACGCTACATCTGCTGGGAACTCTGCTTCCGCTGCGCATCAATCTGAGGTAAACGCTGAGAACTCTGCCACAGCATCCGCTAACTCTGCTCATTTGGCAGAACAGCAAGCAGAC  >  NC_001604.1/34797‑35394`  

                                                                                                                                                                                                                                                                                                          `|`                                                                                                                                                                                                                                                                                                             
      `gACAAAGGCTTGGGGTCCAGCCGATGGCTACACGACCATCGAGTTACGTCGAGTAACCTCCACTACCGACCGATTGGTTGACTTTACGGATGGTTCAATCCTCCGCGCGTATGACCTTAACGTCGCTCAGATTCAAACGATGCACGTAGCGGAAGAGGCCCGTGACCTCACTGCGGATACTATCGGTGTCAATAACGATGGTCACTTGGATGCTCGTGGTCGTCGAATTGTGAACCTAGCGAACGCCGTGGATGACCGCGATGCTGTTCCGTTTGGTCAACTAAAGACCATGGActgtctc                                                                                                                                                                                                                                                                                                     >  1:646047/1‑295 (MQ=255)`  
      `gACAAAGGCTTGGGGTCCAGCCGATGGCTACACGACCATCGAGTTACGTCGAGTAACCTCCACTACCGACCGATTGGTTGACTTTACGGATGGTTCAATCCTCCGCGCGTATGACCTTAACGTCGCTCAGATTCAAACGATGCACGTAGCGGAAGAGGCCCGTGACCTCACTGCGGATACTATCGGTGTCAATAACGATGGTCACTTGGATGCTCGTGGTCGTCGAATTGTGAACCTAGCGAACGCCGTGGATGACCGCGATGCTGTTCCGTTTGGTCAACTAAAGACCATGGACCAGAAc                                                                                                                                                                                                                                                                                                     <  1:270373/301‑1 (MQ=255)`  
            `ggCTTGGGGTCCAGCCGATGGCTACACGACCATCGAGTTACGTCGAGTAACCTCCACTACCGACCGATTGGTTGACTTTACGGATGGTTCAATCCTCCGCTCGTATGACCTTAACGTCGCTCAGATTCAAACGATGCACGTATCGGAAGAGGCCCGTGACCTCACTGCGGATACTATCGGTGTCAATAACGATGGTCACTTGGATGCTCGTGGTCGTCGAATTGTTAACCTAGCGAACGCCGTGGATGACCGCGATGCTGTTCCGTTTGGTCAACTAAGGACCATGGACCAGAACTCAttg                                                                                                                                                                                                                                                                                               >  1:353085/1‑299 (MQ=255)`  
                  `gggTCCAGCCGATGGCTACACGACCATCGAGTTACGTCGAGTAACCTCCACTACCGACCGATTGGTTGACTTTACGGATGGTTCAATCCTCCGCGCGTATGACCTTAACGTCGCTCAGATTCAAACGATGCACGTAGCGGAAGAGGCCCGTGACCTCACTGCGGATACTATCGGTGTCAATAACGATGGTCACTTGGATGCTCGTGGTCGTCGAATTGTGAACCTAGCGAACGCCGTGGATGACCGCGATGCTGTTCCGTTTGGTCAACTAAAGACCATGGACCAGAACTCCTTGCAAGCa                                                                                                                                                                                                                                                                                         >  1:398572/1‑301 (MQ=255)`  
                        `aGCCGATGGCTACACGACCATCGAGTTACGTCGAGTAACCTCCACTACCGACCGATTGGTTGACTTTACGGATGGTTCAATCCTCCGCGCGTATGACCTTAACGTCGCTCAGATTCAAACGATGCACGTATCGGAAGAGGCCCGTGACCTCACTGCGGATACTATCGGTGTCAATAACGATGGTCACTTTGATGCTCGTGGTCGTCGAATTGTGAACCTAGCGAACGCCGTGGATGACCGCGATGCTGTTCCGTTTGGTCAACTAAAGACCATGGACCAGAACTCATGGCAAGCACGTAAt                                                                                                                                                                                                                                                                                   >  1:26415/1‑301 (MQ=255)`  
                          `ccGATGGCTACCCGACCATCGAGTTACGTCGAGTAACCTCCACTACCGACCGATTGGTTGACTTTACGGATGGTTCAATCCTCCGCGCGTATGACCTTAACGTCGCTCAGATTCAAACGATGCACGTAGCGGAAGAGGCCCGTGACCTCACTGCGGATACTATCGGTGTCAATAACGATGGTCACTTGGATGCTCGTGGTCGTCGAATTGTGAACCTAGCGAACGCCGTGGATGACCGCGATGCTGTTCCGTTTGGTCAACTAAAGACCATGGACCAGAACTCATGGCAAGCACGTAATGa                                                                                                                                                                                                                                                                                 <  1:166346/301‑1 (MQ=255)`  
                          `ccGATGGCTACACGACCATCGAGTTACGTCGAGTAACCTCCACTACCGACCGATTGGTTGACTTTACGGATGGTTCAATCCTCCGCGCGTATGACCTTAACGTCGCTCAGATTCAAACGATGCACGTAGCGGAAGAGGCCCGTGACCTCACTGCGGATACTATCGGTGTCAATAACGATGGTCACTTGGATGCTCGTGGTCGTCGAATTGTGAACCTAGCGAACGCCGTGGATGACCGCGATGCTGTTCCGTTTGGTCAACTAAAGACCATGGACCAGAACTCATGGCAAGCACGTAATGa                                                                                                                                                                                                                                                                                 <  1:17507/301‑1 (MQ=255)`  
                             `aTGGCTCCACGACCATCGAGTTACGTCGAGTAACCTCCACTACCGACCGATTGGTTGACTTTACGGATGGTTCAATCCTCCGCGCGTATGACCTTAACGTCGCTCAGATTCAAACGATGCACGTAGCGGAAGAGGCCCGTGACCTCACTGCGGATACTATCGGTGTCAATAACGATGGTCACTTGGATGCTCGTGGTCGTCGAATTGTGAACCTAGCGAACGCCGTGGATGACCGCGATGCTGTTCCGTTTGGTCAACTAAAGACCATGGACCAGAACTCATGGCAAGCACGTAATGAAGc                                                                                                                                                                                                                                                                              <  1:741234/301‑1 (MQ=255)`  
                             `aTGGCTACACGACCATCGAGTTACGTCGAGTAACCTCCACTACCGACCGATTGGTTGACTTTACGGATGGTTCAATCCTCCGCGCGTATGACCTTAACGTCGCTCAGATTCAAACGATGCACGTAGCGGAAGAGGCCCGTGACCTCACTGCGGATACTATCAGTGTCAATAACGATGGTCACTTGGATGCTCGTGGTCGTCGAATTGTGAACCTAGCGAACGCCGTGGATGACCGCGATGCTGTTCCGTTTGGTCAACTAAAGACCATGGACCAGAACTCATGGCAAGCACGTAATGAAGc                                                                                                                                                                                                                                                                              <  1:453807/301‑1 (MQ=255)`  
                               `ggCTACACGACCATCGAGTTACGTCGAGTAACCTCCACTACCGACCGATTGGTTGACTTTACGGATGGTTCTATCCTCCGCGCGTATGACCTTAACGTCGCTCAGATTCAAACGATGCACGTAGCGGAAGAGGCCCGTGACCTCACTGCGGATACTATCGGTGTCAATAACGATGGTCACTTGGATGCTCGTGGTAGTCGAATTGTGAACCTAGCGAACGCCGTGGATGACCGCGATGCTGTTCCGTTTGGTCAACTAAAGACCATGGACCAGAACTCATGGCAAGCACGTAATGAAGCCt                                                                                                                                                                                                                                                                            >  1:341014/1‑301 (MQ=255)`  
                                    `cacGACCATCGGTTTACGTCGAGTAACCTCCACTACCGACCGATTGGTTGACTTTACGGATGGTTCAATCCTCCGCGCGTATGACCTTAACGTCGCTCAGATTCAACCGATGCACGTAGCGGAAGAGGCCCGTGACCTCACTGCGGATACTATCGGTGTCAATAACGATGGTCACTTGGATGCTCGTGGTCGTCGAATTGTGAACCTAGCGAACGCCGTGGATGACCGCGATGCTGTTCCGTTTGGTCAACTAAAGACCATGGACCAGAACTCATGGCAAGCACGTAATGAAGCCTTACAg                                                                                                                                                                                                                                                                       <  1:810579/301‑1 (MQ=255)`  
                                    `cacGACCATCGAGTTACGTCGAGTAACCTCCACTACCGCCCGATTGGTTGACTTTACGGATGGTTCAATCCTCCGCGCGTATGTCCTTAACGTCGCTCAGATTCAAACGATGCACGTAGCGGAAGAGGCCCGTGACCTCACTGCGGATACTATCGGTGTCAATAACGATGGTCACTTGGATGCTCGTGGTCGTCGAATTGTGAACCTAGCGAACGCCGTGGATGACCGCGATGCTGTTCCGTTTGGTCAACTAAAGACCATGGACCAGAACTCATGGCAAGCACGTAATGAAGCCTTACAg                                                                                                                                                                                                                                                                       <  1:546932/301‑1 (MQ=255)`  
                                    `aacGACCATCGAGTTACGTCGAGTAACCTCCACTACCGACCGATTGGTTGACTGTACGGATGGTTCAATCCTCCGCGCGTATGACCTTAACGTCGCTCAGATTCAAACGAAGCACGTAGCGGAAGAGGCCCGTGACCTCACTGCGGATACTATCGGTGTCAATAACGATGGTCACTTGGATGCTCGTGGTCGTCGAATTGTGAACCTAGCGAACGCCGTGGATGACCGCGATGCTGTTCCGTTTGGTCAACTAAAGACCATGGACCAGAACTCATGGCAAGCACGTAATGAAGCCTTACAg                                                                                                                                                                                                                                                                       <  1:200357/300‑1 (MQ=255)`  
                                        `aCCATCGAGTTACGTCGAGTAACCTCCACTACCGACCGATTGGTTGACTTTACGGATGGTTCAATCCTCCGCGCGTATGACCTTAACGTCGCTCAGATTCAAACGATGCACGTAGCGGAAGAGGCCCGTGACCTCACTGCGGATACTATCGGTGTCAATAACGATGGTCACTTGGATGCTCGTGGTCGTCGAATTGTGAACCTAGCGAACGCCGTGGATGACCGCGATGCTTTTCCGTTTGGTCAACTAAAGACCATGGACCAGAACTCATGGCAAGCACGTCATGAAGCCTTACAGTTcc                                                                                                                                                                                                                                                                   >  1:583033/1‑301 (MQ=255)`  
                                         `ccATCGAGTTACGTCGAGTAACCTCCACTACCGACCGATTGGTTGACTTTACGGATGGTTCAATCCTCCGCGCGTATGACCTTAACGTCGCTCAGATTCAAACGATGCACGTAGCGGAAGAGGCCCGTGACCTCACTGCGGATACTATCGGTGTCAATAACGATGGTCACTTGGATGCTCGTGGTCGTCGAATTGTGAACCTAGCGAACGCCGTGGATGACCGCGATGCTGTTCCGTTTGGTCAACTAAAGACCATGGACCAGAACTCATGGCAAGCACGTAATGAAGCCTTACAGTTCCg                                                                                                                                                                                                                                                                  >  1:100296/1‑301 (MQ=255)`  
                                          `cATCGAGTTACGTCGAGTAACCTCCACTACCGACCGATTGGTTGACTTTACGGATGGTTCAATCCTCCGCGCGTATGACCTTAACGTCGCTCAGATTCAAACGATGCACGTAGCGGAAGAGGCCCGTGACCTCACTGCGGATACTATCGGTGTCAATAACGATGGTCACTTGGATGCTCGTGGTCGTCGAATTGTGAACCTAGCGAACGCCGTGGATGACCGCGATGCTGTTCCGTTTGGTCAACTAAAGACCATGGACCAGAACTCATGGCAAGCACGTAATGAAGACTTACAGTTCCGt                                                                                                                                                                                                                                                                 <  1:69578/301‑1 (MQ=255)`  
                                          `cATCGAGTTACGTCGAGTAACCTCCACTACCGACCGATTGGTTGAATTTACGGATGGTTCAATCCTCCGCGCGTATGACCTTAACGTCGCTCAGATTCAAACGATGCACGTAGAGGAAGAGGCCCGTGACCTCACTGCGGATACTATCGGTGTCAATAACGATGGTCACTTGGATGCTCGTGGTCGTCGAATTGTGAACCTAGCGAACGCCGTGGATGACCGCGATGCTGTTCCGTTTGGTCAACTAAAGACCATGGACCAGAACTCATGGCAAGCACGTAATGAAGCCTTACAGTTCCGt                                                                                                                                                                                                                                                                 <  1:636386/301‑1 (MQ=255)`  
                                            `tCGAGTTACGTCGAGTAACCTCCCCTCCCGACCGATTGGTTGACTTTACGGATGGTTCAATCCTCCGCGCGTATGACCTTAACGTCGCTCAGATTCAAACGATGCACGTAGCGGAAGAGGCCCGTGACCTCACTGCGGATACTATCGGTGTCAATAACGATGGTCACTTGGATGCTCGTGGTCGTCGAATTGTGAACCTAGCGAACGCCGTGGATGACCGCGATGCTGTTCCGTTTGGTCAACTAAAGACCATGGACCAGAACTCATGGCAAGCACGTAATGAAGCCTTACAGTTCCGTaa                                                                                                                                                                                                                                                               <  1:686838/301‑1 (MQ=255)`  
                                            `tCGAGTTACGTCGAGTAACCTCCACTACCGACCGATTGGTTGACTTTACGGATGGTTCAATCCTCCGCGCGTATGACCTTAACGTCGCTCAGATTCAAACGATGCACGTAGCGGAAGAGGCCCGTGACCTCACTGCGGATACTATCGGTGTCAATAACGATGGTCACTTGGATGCTCGTGGTCGTCGAATTGTGAACCTAGCGAACGACGTGGATGACCGCGATGCTGTTCCGTTTGGTCAACTAAAGACCATGGACCAGAACTCATGGCAAGCACGTAATGAAGCCTTACAGTTCCGTaa                                                                                                                                                                                                                                                               <  1:671315/301‑1 (MQ=255)`  
                                              `gAGTTACGTCGAGTAACCTCCACTACCGACCGATTGGTTGACTTTACGGATGGTTCAATCCTCCGCGCGTATGACCTTAACGTCGCTCAGATTCAAACGATGCACGTAGCGGAAGAGGCCCGTGACCTCACTGCGGATACTATCGGTGTCAATAACGATGGTCACTTGGATGCTCGTGGTCGTCGAATTGTGAACCTAGCGAACGGCGTGGATGACCGCGATGCTGTTCCGTTTGGTCAACTAAAGACCATGGACCAGAACTCATGGCAAGCACGTAATGAAGCCTTACAGTTCCGTAATg                                                                                                                                                                                                                                                             <  1:404522/301‑1 (MQ=255)`  
                                              `gAGTTACGTCGAGTAACCTCCACTACCGACCGATTGGTTGACTTTACGGATGGTTCAATCCTCCGCGCGTATGACCTTAACGTCGCTCAGATTCAAACGATGCACGTAGCGGAAGAGGCCCGTGACCTCACTGCGGATACTATCGGTGTCAATAACGATGGTCACTTGGATGCTCGTGGTCGTCGAATTGTGAACCTAGCGAACGCCGTGGATGACCGCGATGCTGTTCCGTTTGGTCAACTAAAGATCATGGACCAGAACTCATGGCAAGCACGTAATGAAGCCTTACAGTTCCGTAATg                                                                                                                                                                                                                                                             >  1:567593/1‑301 (MQ=255)`  
                                              `gAGTTACGTCGAGTAACCTCCACTACCGACCGATTGGTTGACTTTACGGATGGTTCAATCCTCCGCGCGTATGACCTTAACGTCGCTCAGATTCAAACGATGCACGTAGCGGAAGAGGCCCGTGACCTCACTGCGGATACTATCGGTGTCAATAACGATGGTCACTTGGATGCTCGTGGTCGTCGAATTGTGAACCTAGCGAACGCCGTGGATGACCGCGATGCTGTTCCGTTTGGTCAACTAAAGACCATGGACCAGAATTCATGGCAAGCACGTAATGAAGCCTTACAgctgtctctta                                                                                                                                                                                                                                                             >  1:726157/1‑291 (MQ=255)`  
                                              `gAGTTACGTCGAGTAACCTCCACTACCGACCGATTGGTTGACTTTACGGATGGTTCAATCCTCCGCGCGAATGACCTTAACGTCGCTCAGATTCAAACGATGCACGTAGCGGAAGAGGCCCGTGACCTCACTGCGGATACTATCGGTGTCAATAACGATGGTCACCTTGATGCTCGTGGTCGTCGAATTGTGAACCTAGCGAACGCCGTGGATGACCGCGATGCTGTTCCGTTTGGTCAACTAAATACCATGGACCAGAACTCATGTCAAGCACGTAATGAAGCCTTACAGTTCCGTAAtt                                                                                                                                                                                                                                                             >  1:116526/1‑300 (MQ=255)`  
                                               `aGTTACGTCGAGTAACCTCCACTACCGACCGATTGGTTGACTTTACGGATGGTTCAATCCTCCGCGCGTATGACCTTAACGTCGCTCAGATTCAAACGATGCACGTAGCGGAAGAGGCCCGTGACCTCACTGCGGATACTATCGGTGTCAATAACGATGGTCACTTGGATGCTCGTGGTCGTCGAATTGTGAACCTAGCGAACGCCGTGGATGACCGCGATGCTGTTCCGTTTGGTCAACTAAAGACCATGGACCAGAACTCATGGCAAGCACGTAATGAAGCCTTACAGTTCCGTAATGa                                                                                                                                                                                                                                                            <  1:484664/301‑1 (MQ=255)`  
                                                       `cGAGTAACCTCCACTACCGACCGATTGGTTGACTTTACGGATGGTTCAATCCTCCGCGCGTATGACCTTAACGTCGCTCAGATTCAAACGATGCACGTAGCGGAAGAGGCCCGTGACCTCACTGCGGATACTATCGGTGTCAATAACGATGGTCACTTGGATGCTCGTGGTCGTCGAATTGTGAACCTAGCGAACGCCGTGGATGACCGCGATGCTGTTCCGTTTGGTCAACTAAAGACCATGGACCAGAACTCGTGGCAAGCACGTAATGAAGCCTTACAGTTCCGGAATGactgtccct                                                                                                                                                                                                                                                    >  1:506631/1‑293 (MQ=255)`  
                                                       `cGAGTAACCTCCACTACCGACCGATTGGTTGACTTTACGGATGGTTCAATCCTCCGCGCGTATGACCTTAACGTCGCTCAGATTCAAACGATGCACGTAGCGGAAGAGGCCCGTGACCTCACTGCGGATACTATCGGTGTCAATAACGATGGTCACTTGGATGCTCGTGGTCGTCGAATTGTGAACCTAGCGAACGCCGTGGATGACCGCGATGCTGTTCCGTTTGGTCAACTAAAGACCATGGACCAGAACTCATGGCAAGCACGTAATGAAGCCTTACAGTTcctgtctcttatacaca                                                                                                                                                                                                                                                    >  1:528784/1‑286 (MQ=255)`  
                                                        `gAGTAACCTCCACTACCGACCGATTGGTTGACTTTACGGATGGTTCAATCCTCCGCGCGTATGACCTTAACGTCGCTCAGATTCAAACGATGCACGTAGCGGAAGAGGCCCGTGACCTCACTGCGGATACTATCGGTGTCAATAACGATGGTCACTTGGATGCTCGTGGTCGTCGAATTGTGAACCTAGCGAACGCCGTGGATGACCGCGATGCTGTTCCGTTTGGTCAACTAAAGACCATGGACCAGAACTCATGGCAAGCACGTAATGAAGCCTTACAGTTCCGTAATGAGGCTGAGAc                                                                                                                                                                                                                                                   <  1:513302/301‑1 (MQ=255)`  
                                                            `aaCCTCCACTACCGACCGATTGGTTGACTTTACGGATGGTTCAATCCTCCGCGCGTATGACCTTAACGTCGCTCAGATTCAAACGATGCACGTAGCGGAAGAGGCCCGTGACCTCACTGCGGATACTATCGGTGTCAATAACGATGGTCACTTGGATGCTCGTGGTCGTCGAATTGTGAACCTAGCGAACGCCGTGGATGACCGCGATGCTGTTCCGTTTGGTCAACTAAAGACCATGGACCAGAACTCATGGCAAGCACGTAATGAAGCCTTACAGTTCCGTAATGAGGCTGAGACTTTc                                                                                                                                                                                                                                               >  1:703448/1‑301 (MQ=255)`  
                                                              `ccgcgaCTACCGAACGATTGGTTGACTTTACGGATGGTTCAAGCCTCCGCGCGTATGACCTTAACGTCGCTCAGATTCAAACGATGCACGTAGCGGAAGAGGCCCGTGACCTCACTGCGGATACTATCGGTGTCAATAACGATGGTAACTTGGATGCTCGTGGTCGTCGAATTGTGAACCTAGCGAACGCCGTGGATGACCGCGATGCTGTTCCGTTTGGTCAACTAAAGACCATGGAACAGAACTCATGGAAAGCACGTAATGAAGCCTTACAGTTCCGTAATGAGGCTGAGACTTTCAg                                                                                                                                                                                                                                             <  1:781599/296‑1 (MQ=255)`  
                                                               `ctctACTACCGACCGATTGGTTGACTTTACGGATGGTTCAATCCTCCGCGCGTATGACCTTAACGTCGCTCATATTCAAACGATGCACGTAGCGGAAGAGGCCCGTGACCTCACTGCGGATACTATCGGTGTCAATAACGATGGTCACTTGGATGCTCGTGGTCGTCGAATTGTGAACCTAGCGAACGCCGTGGATGACCGCGATGCTGTTCCGTTTGGTCAACTAAAGACCATGGACCAGAACTCATGGCAAGCACGTAATGAAGCCTTACAGTTCCGTAATGAGGCTGAGACTTTCAGa                                                                                                                                                                                                                                            >  1:494087/1‑301 (MQ=255)`  
                                                               `cTCCACTACCGACCGATTGGTTGACTTTACGGATGGTTCAATCCTCCGCGCGTATGACCTTAACGTCGCTCAGATTCAAACGATGCACGTAGCGGAAGAGGCCCGTGACCTCACTGCGGATCCTATCGGTGTCACTAACGATTGTCACTTTGATGCTCGTTGTCGTCGAATTGTGAACCTAGCGAACGCCGTGGATGACCGCGATGCTGTTCCGTTTGTTCAACTAAAGACCATGGACCAGAACTCATGGCCAGCACGTAATGAAGCCTTACAGTTCCGTAATGCGGCTGAGACTTTCCGa                                                                                                                                                                                                                                            >  1:574376/1‑301 (MQ=255)`  
                                                               `cTCCACTACCGACCGATTGGTTGACTTTACGGATGGTTCAATCCTCCGCGCGTATGACCTTAACGTCGCTCAGATTCAAACGATGCACGTAGCGGAAGAGGCCCGTGACCTCACTGCGGATACTATCGGTGTCAATAACGATGGTCACTTGGATGCTCGTGGTCGTCGAATTGTGAACCTAGCGAACGCCGTGGATGACCGCGATGCTGTTCCGTTTGGTCAACTAAAGACCATGGACCAGAACTCATGGCAAGCACGTAATGAAGCCTTACAGTTCCGTaactgtctcttatacacatct                                                                                                                                                                                                                                            >  1:621414/1‑282 (MQ=255)`  
                                                                `cccACTACCGACCGATTGGTTGACTTTACGGATGGTTCAATCCTCCGCGCGTATGACCTTAACGTCGCTCAGATTCAAACGATGCACGTAGCGGAAGAGGCCCGTGACCTCACTGCGGATACTATCGGTGTCAATAACGATTGTCACTTGGATGCTCGTGGTCGTCGAATTGTGAACCTAGCGAACGCCGTGGATGACCGCGATGCTGTTCCGTTTGGTCAACTAAAGACCATGGACCAGAACTCATGGCAAGCACGTAATGAAGCCTTACAGTTCCGTAATGAGGCTGAGACTTTCAGaa                                                                                                                                                                                                                                           <  1:559366/300‑1 (MQ=255)`  
                                                                 `ccACTACCGACCGATTGGTTGACTTTACGGATGGTTCAATCCTCCGCGCGTATGACCTTAACGTCGCTCAGATTCAAACGATGCACGTAGCGGAAGAGGCCCGTGACCTCACTGCGGATACTATCGGTGTCAATAACGATGGTCACTTGGATGCTCGTGGTCGTCGAATTGTGAACCTAGCGAACGCCGTGGATGACCGCGATGCTGTTCCGTTTGGTCAACTAAAGACCATGGACCAGAACTCATGGCAAGCACGTAATGAAGCCTTCCAGTTCCGTAATGAGGCTGAGACTTTCAGaaa                                                                                                                                                                                                                                          >  1:221657/1‑301 (MQ=255)`  
                                                                   `aCTACCGACCGATTGGTTGACTTTACGGATGGTTCAATCCTCCGCGCGTATGACCTTAACGTCGCTCAGATTCAAACGATGCACGTAGCGGAAGAGGCCCGTGACCTCACTGCGGATACTATCGGTGTCAATAACGATGGTCACTTGGATGCTCGTGGTCGTCGAATTGTGAACCTAGCGAACGCCGTGGATGACCGCGATGCTGTTCCGTTTGGTCAACTAAAGACCATGGACCAGAACTCATGGCAAGCACGTAATGAAGCCTTACAGTTCCGTAATGAGGCTGAGACTTTCAGAAAcc                                                                                                                                                                                                                                        <  1:298330/301‑1 (MQ=255)`  
                                                                    `cTACCGACCGATTGGTTGACTTTACGGATGGTTCAATCCTCCGCGCGTATGACCTTAACGTCGCTCAGATTCAAACGATGCACGTAGCGGAAGAGGCCCGTGACCTCACTGCGGATACTATCGGTGTCAATAACGATGGTCCCTTGGATGCTCGTGGTCGTCGAATTGTGAACCTAGCGAACGCAGTGGATGACCGCGATGCTGTTCCGTTTGGTAAACTAAAAACCATGGACAAGAACTAATGGACAGCAAGTAATGAAGCATTACATATCCGTACTGAAGCTGAGAATTACAGAAAACa                                                                                                                                                                                                                                       >  1:499801/1‑301 (MQ=255)`  
                                                                            `cgaTTGGTTGACTTTACGGATGGTTCAAACCTCCGCGCGTATGACCTTAACGTCGCTCAGATTCAAACGATGCACGTAGCGGAAGAGGCCCGTGACCTCACTGCGGATACTATCGGTGTCAATAACGATGGTCACTTGGATGCTCGAGGTCGTCGAATTGTGAACCTAGCGAACGACGTGGATGACCGCGATGCTGTTCCGTTTGGTCAACTAAAGACCATGGACCAGAACTCATGGCAAGCACGTAATGAAGCCTTACAGTTCCGTAATGAGGCTGAGACTTTCAGAAACCAAGCGGAgg                                                                                                                                                                                                                               <  1:230373/301‑1 (MQ=255)`  
                                                                                     `tgggctcggagatgtgtataagagacagcgTATGACCTTAACGTCGCTCAGATTCAAACGATGCACGTAGCGGAAGAGGCCCGTGACCTCACTGCGGATACTATCGGTGTCAATAACGATGGTCACTTGGATGCTCGTGGTCGTCGAATTGTGAACCTAGCGAACGCCGTGGATGACCGCGATGCTGTTCCGTTTGGTCAACTAAAGACCATGGACCAGAACTCATGGCAAGCACGTAATGAAGCCTTACAGTTCCGTAATGAGGCTGAGACTTTCAGAAACCAAGCGGAGGGCTTTAAGa                                                                                                                                                                                                                      <  1:34674/274‑1 (MQ=255)`  
                                                                                       `cTTTACGGATGGTTCAATCCTCCGCGCGTATGACCTTAACGTCGCTCAGATTCAAACGATGCACGTAGCGGAAGAGGCCCGTGACCTCACTGCGGATACTATCGGTGTCAATAACGATGGTCACTTGGATGCTCGTGGTCGTCGAATTGTGAACCTAGCGAACGCCGTGGATGACCGCGATGCTGTTCCGTTTGGTCAACTAAAGACCATGGACCAGAACTCATGGCAAGCACGTAATGAAGCCTTACAGTTCCGTAATGAGGCTGAGACTTTCAGAAACCAAGCGGAGGGCTTTAAgctg                                                                                                                                                                                                                    >  1:391674/1‑298 (MQ=255)`  
                                                                                            `cGGATGGTTCAATCCTCCGCGCGGATGACATTAACGTCGCTCAGATTCAAACGATGCACGTAGCGGAAGAGGCCCGTGACCTCACTGCGGATACTATCGGTGTCAATAACGATGGTCACTTGGATGCTCGTGGTCGTCGAATGGTGAACCTAGCGAACGCCGTGGATGACCGCGATGCTGTTCCGTTTGGTCAACTAAAGACCATGGACCAGAACTCATGGCAAGCACGTAATGAAGCCTTACAGTTCCGTAATGAGGCTGAGACTTTCAGAAACCAAGCGGAGGGCTTTAAGAACGAGTc                                                                                                                                                                                                               <  1:330808/301‑1 (MQ=255)`  
                                                                                                `tGGTTCAATCCTCCGCGCGTATGACCTTAACGTCGCTCAGATTCAAACGATGCACGTAGCGGAAGAGGCCCGTGACCTCACTGCGGATACTATCGGTGTCAATAACGATGGTCACTTGGATGCTCGTGGTCGTCGAATTGTGAACCTAGCGAACGCCGTGGATGACCGCGATGCTGTTCCGTTTGGTCAACTAAAGACCATGGACCAGAACTCATGGCAAGCACGTAATGAAGCCTTACAGTTCCGTAATGAGGCTGAGACTTTCAGAAACCAAGCGGAGGGCTTTAAGAACGAGGccctg                                                                                                                                                                                                           >  1:427001/1‑298 (MQ=255)`  
                                                                                                  `gTTCAATCCTCCGCGCGTATGACCTTAACGTCGCTCAGATTCAAACGATGCACGTAGCGGAAGAGGCCCGTGACCTCACTGCGGATACTATCGGTGTCAATAACGATGGTCACTTGGATGCTCGTGGTCGTCGAATTGTGAACCTAGCGAACGCCGTGGATGACCGCGATGCTGTTCCGTTTGGTCAACTAAAGACCATGGACCAGAACTCATGGCAAGCACGTAATGAAGCCTTACAGTTCCGTAATGAGGCTGAGACTTTCAGAAACCAAGCGGAGGGCTTTAAGactgtctcttataa                                                                                                                                                                                                         >  1:803499/1‑288 (MQ=255)`  
                                                                                                  `gTTCAATCCTCCGCGCGTATGACCTTAACGTCGCTCAGATTCAAACGATGCACGTAGCGGAAGAGGCCCGTGACCTCACTGCGGATACTATCGGTGTCAATAACGATGGTCACTTGGATGCTCGTGGTCGTCGAATTGTGAACCTAGCGAACGCCGTGGATGACCGCGATGCTGTTCCGTTTGGTCAACTAAAGACCATGGACCAGAACTCATGGCAAGCACGTAATGAAGCCTTACAGTTCCGTAATGAGGCTGAGACTTTCAGAAACCAAGCGGAGGGCTTTAAGAACGAGTCCAGTAc                                                                                                                                                                                                         >  1:717335/1‑301 (MQ=255)`  
                                                                                                  `gTTCAATCCTCCGCGCGTATGACCTTAACGTCGCTCAGATTCAAACGATGCACGTAGCGGAAGAGGCCCGTGACCTCACTGCGGATACTATCGGTGTCAATAACGATGGTCACTTGGATGCTCGTGGTCGTCGAATTGTGAACCTAGCGAACGCCGTGGATGACCGCGATGCTGTTCCGTTTGGTCAACTAAAGACCATGGACCAGAACTCATGGCAAGCACGTAATGAAGCCTTACAGTTCCGTAATGAGGCTGAGACTTTCAGAAACCAAGCGGAGGGCTTTAAGAACGAGTCCAGTAc                                                                                                                                                                                                         <  1:710679/301‑1 (MQ=255)`  
                                                                                                  `gTTCAATCCTCCGCGCGTATGACCTTAACGTCGCTCAGATTCAAACGATGCACGTAGCGGAAGAGGCCCGTGACCTAACTGCGGATACTATCGGTGTCAATAACGATGGTCACTTGGATGCTCGGGGTCGTCGAATTGTGAACCTAGCGAACGCCGTGGATGACCGCGATGCTGTTCCGTTTGGTCAACTAAAGACCATGGACCAGAACTCATGGCAAGCACGTAATGAAGCCTTACAGTTCCGTAATGAGGCTGAGACTTTCAGAAACCAAGCGGAGGGCTTTAAGAACGAGTCCAGTAc                                                                                                                                                                                                         <  1:775009/301‑1 (MQ=255)`  
                                                                                                  `gTTCAATCCTACGCGCGTATGACCTTAACGTCGCTCAGATTCAAACGATGCACGTAGCGGAAGAGGCCCGTGACCTCACTGCGGATACTATCGGTGTCAATAACGATGGTCACTTGGATGCTCGTGGTCGTCGAATTGTGAACCTAGCGAACGCCGTGGATGACCGCGATGCTGTTCCGTTTGGTCAACTAAAGACCATGGACCAGAACTCATGGCAAGCACGTAATGAAGCCTTACAGTTCCGTAATGAGGCTGAGACTTTCAGGAACCCAGCGGAGGGCTTTAAGAACGAGTCCAGTAc                                                                                                                                                                                                         >  1:816856/1‑301 (MQ=255)`  
                                                                                                  `gTTCAATCCGCCGCGCGTATGACCTTAACGTCGCTCAGATTCAAACGATGCACGTAGCGGAAGAGGCCCGTGACCTCACTGCGGATACTATCGGTGTCAATAACGATGGTCACTTGGATGCTCGTGGTCGTCGAATTGTGAACCTAGCGAACGCCGTGGATGACCGCGATGCTGTTCCGTTTGGTCAACTAAAGACCATGGACCAGAACTAATGGCAAGCACGTAATGAAGCCTTACAGTTCCGTAATGAGGCTGAGACTTTCAGAAACCAAGCGGAGGGCTTTAAGAACGAGTCCAGTAc                                                                                                                                                                                                         <  1:411901/301‑1 (MQ=255)`  
                                                                                                  `atgtgtataagagacagTATGACCTTAACGTCGCTCAGATTCAAACGATGCACGTAGCGGAAGAGGCCCGTGACCTCACTGCGGATACTATCGGTGTCAATAACGATGGTCACTTGGATGCTCGTGGTCGTCGAATTGTGAACCTAGCGAACGCCGTGGATGACCGCGATGCTGTTCCGTTTGGTCAACTAAAGACCATGGACCAGAACTCATGGCAAGCACGTAATGAAGCCTTACAGTTCCGTAATGAGGCTGAGACTTTCAGAAACCAAGCGGAGGGCTTTAAGAACGAGTCCAGTAc                                                                                                                                                                                                         <  1:589075/285‑1 (MQ=255)`  
                                                                                                   `ttCAATCCTCCGCGCGTATGACCTTAACGTCGCTCAGATTCAAACGATGCACGTAGCGGAAGAGGCCCGTGACCTCACTGCGGATACTATCGGTGTCAATAACGATGGTCACTTGGATGCTCGTGGTCGTCGAATTGTGAACCTAGCGAACGCCGTGGATGACCGCGATGCTATTCCGTTTGGTCAACTAAAGACCATGGACCAGAACTCATGGCAAGCACGTAATGAAGCCTTACAGTTCCGTAATGAGGCTGAGACTTTCAGAAACCAAGCGGAGGGCTTTAAGAACGAGTCCAGtctg                                                                                                                                                                                                        >  1:790314/1‑298 (MQ=255)`  
                                                                                                        `cccagcgcgcgTATGACCTTAACGTCGCTCAGATGCAAACGATGCACGTAGCGGAAGAGGCCCGGGACCTCACGGCGGATACTATCGGTGTCAATAACGATGGTCATTTGGATGCTCGTGGTCGTCGAATTGTGAACCTAGCGAACGCCGTGGATGACCGCGATGCTGTTCCGTTTGGTCAACTAAAGACCATGGACAAGAACTCATGGCAAGCACGTAATGAAGCCTTACAGTTCCGTAATGAGGCTGAGACTTTCAGAAACCAAGCGGAGGGCTTTAAGAACGAGTCCAGTACCAACGc                                                                                                                                                                                                   <  1:852/296‑1 (MQ=255)`  
                                                                                                        `agggacaggcgTATGACCTTAACGTCCCTCAAATTCAAAGGTTGCACGTAGCGGAAGAGGCCCGTGGCCTCACTGCGGATACTATCGGTGTCAATAACGATGGTCACTTGGATGCTCGTGGTCGTCGAATTGTGAACCTAGCGAACGCCGTGGATGACCGCGATGCTGTTCCGTTTGGTCAACTAAAGACCATGGACCAGAACTCATGGCAAGCACGTAATGAAGCCGTACAGTTCCGTAATGAGGCTGAGACTTTCAGAAACCAAGCGGAGGGCTTTAAGAACGAGTCCAGTACCAACGc                                                                                                                                                                                                   <  1:460257/293‑1 (MQ=255)`  
                                                                                                              `gcgcgTATGAACTTAACGTCGCTCAGATTCAAACGATGCACGTAGCGGAAGAGGCCCGTGACCTCACTGCGGATACTATCGGTGTCAATAACGATGGTCACTTGGATGCTCGTGGTCGTCGAATTGTGAACCTAGCGAACGCCGTGGATGACCGCGATGCTGTTCCGTTTGGTCAACTAAAGACCATGGACCAGAACTCATGGCAAGCACGTAATGAAGCCTTACAGTTCCGTAATGAGGCTGAGACTTTCAGAAACCAAGCGGAGGGCTTTAAGAACGAGTCCAGTACCAACGCTACGaa                                                                                                                                                                                             <  1:749817/301‑1 (MQ=255)`  
                                                                                                               `agagtatgaactaaacggaaagaaGATTAAAACGAAGCACGTAGGGAAAGAGGGCCGGGAACTCACTGCGGAAAAGAACGGTGGAAAAAAAGATGGACAGTTGGATGCGCGTGGTAGTCGAATAGTGAAAATAGCGAACGGCGGGGATGACCGAGAGGAAGTTCCGTAAGGGAAAATAAAGAAAAAGGACCAGAAAAAAAGGAAAGCACGAAAAGAAGAAGTAAAGAGGAGTAATGAGGCTGAGACTTTAAGAAACCAAGCGGAGGGCTTTAAGAACGAGTCCAGTACCAACGCTACGAac                                                                                                                                                                                            <  1:369927/278‑1 (MQ=255)`  
                                                                                                                 `cgTATGACCTTAACGTCGCTCAGATTCAAACGATGAACGTAGCGGAAGAGGCCCGTGACCTCACTGCGGATACTATCGGTGTCAATAACGATGGTCACTTGGATGCTCGTGGTCGTCGAATTGTGAACCTAGCGAACGCCGTGGATGACCGCGATGCTGTTCCGTTTGGTCAACTAAAGACCATGGACCAGAACTCATGGCAAGCACGTAATGAAGCCTTACAGTTCCTTAATGAGGCTGAGACTTTCAGAAACCAAGCGGAGGGCTTTAAGAACGAGTCCAGTACCAACGCTACGAacac                                                                                                                                                                                          <  1:433605/301‑1 (MQ=255)`  
                                                                                                                 `cgTATGACCTTAACGTCGCTCAGAGTAAAACGATGCACGGAGCGGAAGAGGCCCGTGACCTCACTGCGGATACTATCGGTGTCAATAACGATGGTCACTTGGATGCTCGTGGTCGTCGAATTGTGAACCTAGCGAACGCCGTGGATGACCGCGATGCTGTTACGTTTGGTCAACTAAAGACCATGGAACAGAACTCATGGCAAGCACGTAATGAAGCCTTACAGTTCCGTAATGAGGCTGAGACTTTCAGAAACCAAGCGGAGGGCTTTAAGAACGAGTCCAGTACCAACGCTACGAacac                                                                                                                                                                                          <  1:796507/301‑1 (MQ=255)`  
                                                                                                                      `gACCTTAACGTCGCTCAGATTCAAACGATGCACGTAGCGGAAGAGGCCCGTGACCTCACTGCGGATACTATCGGTGTCAATAACGATGGTCACTTGGATGCTCGTGGTCGTCGAATTGTGAACCTAGCGAACGCCGTGGATGACCGCGATGCTGTTCCGTTTGGTCAACTAAAGACCATGGACCAGAACTCATGGCAAGCACGTAATGAAGCCTTACAGTTCCGTAATGAGGCTGAGACTTTCAGAAACCAAGCGGAGGGCTTTAAGAACGAGTCCAGTACCAACGCTACGAACACAAAGc                                                                                                                                                                                     <  1:214594/301‑1 (MQ=255)`  
                                                                                                                      `agagacagcGTCGCTCAGATTCAAACGATGCACGTAGCGGAAGAGGCCCGTGACCTCACTGCGGATACTATCGGTGTCAATAACGATGGTCACTTGGATGCTCGTGGTCGTCGAATTGTGAACCTAGCGAACGCCGTGGATGACCGCGATGCTGTTCCGTTTGGTCAACTAAAGACCATGGACCAGAACTCATGGCAAGCACGTAATGAAGCCTTACAGTTCCGTAATGAGGCTGAGACTTTCAGAAACCAAGCGGAGGGCTTTAAGAACGAGTCCAGTACCAACGCTACGAACACAAAGc                                                                                                                                                                                     <  1:654645/293‑1 (MQ=255)`  
                                                                                                                        `ccTTAACGTCGCTCAGATTCAAACGATGCACGTATCGGAAGAGGCCCGTGACCTCACTGCGGATACTATCGGTGTCAATAACGATGGTCACTTGGATGCTCGTGGTCGTCGAATTGTGAACCTAGCGAACGCCGTGGATGACCGCGATGCTGTTCCGTTTGGTCAACTAAAGACCATGGACCAGAACTCATGGCAAGCACGTAATGAAGCCTTACAGTTCCGTAATGAGGCTGAGACTTTCAGAAACCAAGCGGAGGGCTTTAAGAACGAGTCCAGTACCAACGCTACGAAAACAAAGCAg                                                                                                                                                                                   >  1:134947/1‑301 (MQ=255)`  
                                                                                                                           `gtgggctcggagatgtgtataagagacagTAGCGGAAGAGGCCCGTGACCTCACTGCGGATACTATCGGTGTCAATAACGATGGTCACTTGGATGCTCGTGGTCGTCGAATTGTGAACCTAGCGAACGCCGTGGATGACCGCGATGCTGTTCCGTTTGGTCAACTAAAGACCATGGACCAGAACTCATGGCAAGCACGTAATGAAGCCTTACAGTTCCGTAATGAGGCTGAGACTTTCAGAAACCAAGCGGAGGGCTTTAAGAACGAGTCCAGTACCAACGCTACGAACACAAAGCAGTgg                                                                                                                                                                                <  1:376711/273‑1 (MQ=255)`  
                                                                                                                                            `aaaCGATGCACGTAGCGGAAGAGGCCCGTGACCTCCCTGCGGATACTATCGGTGTCAAAAACGATGGTAACTTGGATGCTCGTGGTCGTCGAATTGTGAACCTAGCGAACGCCGTGGATGACCGCGATGCTGTTCCGTTTGGTCAACTAAAGACCATGGACCAGAACTCATGGCAAGCACGTAATGAAGCCTTACAGTTCCGTAATGAGGCTGAGACTTTCAGAAACCAAGCGGAGGGCTTTAAGAACGAGTCCAGTACCAACGCTACGAACACAAAGCAGTGGCGCGATGAGACCAAggg                                                                                                                                                               <  1:677376/301‑1 (MQ=255)`  
                                                                                                                                                    `cACGTAGCGGAAGAGGCCCGTGACCTCACTGCGGATACTATCGGTGTCAATAACGATGGTCACTTGGATGCTCGTGTTCGTCGAATTGTGAACCTAGCGAACGCCGTGGATGACCGCGATGCTGTTCCGTTTGGTCAACTAAAGACCATGGACCAGAACTCATGGCAAGCACGTAATGAATCCTTACAGTTCCGTAATGAGGCTGAGACTTTCAGAAACCAAGCGGAGGGCTTTAAGAACGAGTCCAGTACCAACGCTACGAACACAAAGCAGTGGCGCGATGAGACCAAGGGTTTCCgag                                                                                                                                                       <  1:597812/301‑1 (MQ=255)`  
                                                                                                                                                        `tAGCGGAAGAGGCCCGTGACCTCACTGCGGATACTATCGGTGTCAATAACGATGGTCACTTGGATGCTCGAGGTCGTCGAATTGTGAACCTAGCGAACGCCGTGGATGACCGCGATGCTGTTCCGTTTGGTCAACTAAAGACCATGGACCAGAACTCATGGCAAGCACGTAATGAAGCCTTACAGTTCCGTAATAAGGCTGAAACTTTCAGAAACCAAGCGGAGGGCTTTAAGAAAGAGCCCAGTACCAACGCTACGAACACAAAGCAGTGGCTACATGAGACCACGGGGTTCCGACACGa                                                                                                                                                   >  1:91761/1‑301 (MQ=255)`  
                                                                                                                                                                          `gtgtataagagacagcTATCGGTGTCAATAACGATGGTCACTTGGATGCTCGTGGGCGTCGAATTGTGAACCGAGCGAACGCCGTGGATGACCGCGATGCTGTTCCGTTTGGTCAACTAAAGAACATGGACCAGAACTCATGGCAAGCACGTAATGAAGCCTTACAGTTCCGTAATGAGGCTGAGTCTTTCAGAAACCAAGCGGAGGGCTTTAAGAACGAGTCCAGTACCAACGCTACGAACACAAAGCAGTGGCGCGATGAGACCAAGGGTTTCCGAGACGAAGCCAAGCGGTTCAAGaa                                                                                                                                 <  1:360844/286‑1 (MQ=255)`  
                                                                                                                                                                          `cccccACTGCGGATACTATCGGTGTCAATAACGATGGTCACTTGGATGCTCGTGGTCGTCGAATTGTGAACCTAGCGAACGCCATGGATGACCGCGATGCTGTTCCGTTTGGTCAACTAAAGACCATGGACCAGAACTCATGGCAAGCACGTAATGAAGCCTTACAGTTCCGTAATGAGGCTGAGACTTTCAGAAACCAAGCGGAGGGCTTTAAGAACGAGTCCAGTACCAACGCTACGAACACAAAGCAGTGGCGCGATGAGACCAAGGGTTTCCGAGACGAAGCCAAGCGGTTCAAGaa                                                                                                                                 <  1:154319/297‑1 (MQ=255)`  
                                                                                                                                                                          `acagcACTGCGGATACTATCGGTGTCAATAACGATGGTCACTTGGATGCTCGTGGTCGTCGAATTGTGAACCTAGCGAACGCCGTGGATGACCGCGATGCTGTTCCGTTTGGTCAACTAAAGACCATGGACCAGAACTCATGGCAAGCACGTAATGAAGCCTTACAGTTCCGTAATGAGGCTGAGACTTTCAGAAACCAAGCGGAGGGCTTTAAGAACGAGTCCAGTACCAACGCTACGAACACAAAGCAGTGGCGCGATGAGACCAAGGGTTTCCGAGACGAAGCCAAGCGGTTCAAGaa                                                                                                                                 <  1:314631/297‑1 (MQ=255)`  
                                                                                                                                                                          `aCCTCACTGCGGATACTATCGGTGTCAATAACGATGGTCACTTGGATGCTCGTGGTCGTCGAATTGTGAACCTAGCGAACGCCGTGGATGACCGCGATGCTGTTCCGTTTGGTCAACTAAAGACCATGGACCAGAACTCATGGCAAGCACGTAATGAAGCCTTACAGTTCCGTAATGAGGCTGAGACTTTCAGAAACCAAGCGGAGGGCTTTAAGAACGAGTCCAGTACCAACGCTATGAACACAAAGCAGTGGCGCGATGAGACCAAGGGTTTCCGAGACGAAGCCGAGCGGTTCAAGaa                                                                                                                                 <  1:664012/301‑1 (MQ=255)`  
                                                                                                                                                                          `aCCTAACTGCGGATACTATCGGTGTCAATAACGATGGTCACTTGGATGCTCGTGGTCGTCGAATTGTGAACCTAGCGAACGCCGTGGATGACCGCGATGCTGTTCCGTTTGGTCAACTAAAGACCATGGACCAGAACTCATGGCAAGCACGTAATGAAGCCTTACAGTTCCGTAATGAGGCTGAGACTTTCAGAAACCAAGCGGAGGGCTTTAAGAACGAGTCCAGTACCAACTCTACGAACACAAAGCAGTGGCGCGATGAGACCAAGGGTTTCCGAGACGAAGACAAGCGGTTCAAGaa                                                                                                                                 <  1:245524/301‑1 (MQ=255)`  
                                                                                                                                                                           `ccTCACTGCGGATACTATCGGTGTCAATAACGATGGTCACTTGGATGCTCGTGGTCGTCGAATTGTGAACCTAGCGAACGCCGTGGATGACCGCGATGCTGTTCCGTTTGGTCAACTAAAGACCATGGACCAGAACTCATGGCAAGCACGTAATGAAGCCTTACAGTTCCGTAATGAGGCTGAGACTTTCAGAAACCAAGCGGAGGGCTTTAAGAACGAGTCCAGTACCAACGCTACGAACACAAAGCAGTGGCGCGCTAAGACCAAGGGTTTCCGAGACGAAGCCAAGCGGTTCAAGaaa                                                                                                                                >  1:521320/1‑300 (MQ=255)`  
                                                                                                                                                                           `ccTCACTGCGGATACTATCGGTGTCAATAACGATGGTCACTTGGATGCTCGTGGTCGTCGAATTGTGAACCTAGCGAACGCCGTGGATGACCGCGATGCTGTTCCGTTTGGTCAACTAAAGACCATGGACCAGAACTCATGGCAAGCACGTAATGAAGCCTTACAGTTCCGTAATGAGGCTGAGACTTTCAGAAACCAAGCGGAGGGCTTTAAGAACGAGTCCAGTACCAACGCTACGAACACAAAGCAGTGGCGCGATGAGACCAAGGGTTTCCGAGACGAAGCCAAGCGGTTCAAGaaa                                                                                                                                >  1:824385/1‑300 (MQ=255)`  
                                                                                                                                                                           `ccTCACTGCGGATACTATCGGTGTCAATAACGATGGTCACTTGGATGCTCGTGGTCGTCGAATTGTGAACCTAGCGAACGCCGTGGATGACCGCGATGCTGTTCCGTTTGGTCAACTAAAGACCATGGACCAGAACTCATGGCAAGCACGTAATGAAGCCTTACAGTTCCGTAATGAGGCTGAGACTTTCAGAAACCAAGCGGAGGGCTTTAAGAACGAGTCCAGTACCAACGCTACGAACACAAAGCAGTGGCGCGATGAGACCAAGGGTTTCCGAGACGAAGCCAAGCGGTTCAAGaaa                                                                                                                                >  1:758301/1‑300 (MQ=255)`  
                                                                                                                                                                            `cTCACTGCGGATACTATCGGTGTCAATAACGATGGTCACTTGGATGCTCGTGGTCGTCGAATTGTGAACCTAGCGAACGCCGTGGATGACCGCGATGCTGTTCCGTTTGGTCAACTAAAGACCATGGACCAGAACTCATGGCAAGCACGTAATGAAGCCTTACAGTTCCGTAATGAGGCTGAGACTTTCAGAAACCAAGCGGAGGGCTTTAAGAACGAGTCCAGTACCAACGCTACGAACACAAAGCAGTGGCGCGATGAGACCAAGGGTTTCCGAGACGAAGCCAAGCGGTTCAAGAATa                                                                                                                               >  1:148441/1‑301 (MQ=255)`  
                                                                                                                                                                                `ctgcGGATCCTATCGGTTTCAATACCGATGGTCACTTGGATGCTCGTGGTCGTCGAATTGTGAACCTAGCGAACGCCGTTGATGACCGCGATCCTGTTCCGTTTGGTCAACTAAAGACCATGGACCAGAACTCATGGCAAGCACGTAATGAAGACTTACAGTTCCGTAATGAGGCTGAGACTTTCAGAAACCAAGCGGAGGGCTTTAAGAACGAGTCCAGTACCAACGCTACGAACACAAAGCAGTGGCGCGATGAGACCAAGGGTTTCCGAGACGAAGCCGAGCGGTTCAAGAATACGGc                                                                                                                           <  1:732458/298‑1 (MQ=255)`  
                                                                                                                                                                                `ctgcGGATACTATCGGTGTCAATAACGATGGTCACTTGGATGCTCGTTGTCGTCGAATTGTGAACCTAGCGAACGCCGTGGATGACCGCGATGCTGTTCCGTTTGGTCAACTAAAGACCATGGACCAGAACTCATGGCAAGAACGTAATGAAGCCTTACAGTTCCGTAATGAGGCTGAGACTTTCAGAAACCAAGCGGAGGGCTTTAAGAACGAGTCCAGTACCAACGCTACGAACACAAAGCAGTGGCGCGATGAGACCAAGGGTTTCCGAGACGAAGCCAAGCGGTTCAAGAATACGGc                                                                                                                           >  1:468400/4‑301 (MQ=255)`  
                                                                                                                                                                                `ctgcGGATACTATCGGTGTCAATAACGATGGTCACTTGGATGCTCGTGGTCGTCGAATTGTGAACCTAGCGAACGCCGTGGATGACCGCGATGCTGTTCCGTTTGGTCAACTAAAGACCATGGACCAGAACTCATGGCAAGCACGTAATGAAGCCTTACAGTTCCGTAATGAGGCTGAGACTTTCAGAAACCAAGCGGAGGGCTTTAAGAACGAGTCCAGTACCACCGCTACGAACACAAAGCAGTGGCGCGATGAGACCAAGGGTTTCCGAGACGAAGACGAGCGGTTCAAGAATACGGc                                                                                                                           >  1:291995/4‑301 (MQ=255)`  
                                                                                                                                                                                `ctgcGGATACTATCGGTGTCAATAACGATGGTCACTTGGATGCTCGTGGTCGTCGAATTGTGAACCTAGCGAACGCCGTGGATGACCGCGATGCTGTTCCGTTTGGTCAACTAAAGACCATGGACCAGAACTCATGGCAAGCACGTAATGAAGCCTTACAGTTCCGTAATGAGGCTGAGACTTTCAGAAACCAAGCGGAGGGCTTTAAGAACGAGTCCAGTACCAACGCTACGAACACAAAGCAGTGGCGCGATGAGACCAAGGGTTTCCGAGACGAAGCCAAGCGGTTCAAGAATACGGc                                                                                                                           >  1:122698/4‑301 (MQ=255)`  
                                                                                                                                                                                       `tACTATCGGTGTCAATAACGATGGTCACTTGGATGCTCGTGGTCGTCGAATTGTGAACCTAGCGAACGCCGTGGATGACCGCGATGCTGTTCCGTTTGGTCAACTAAAGACCATGGACCAGAACTCATGGCAAGCACGTAATGAAGCCTTACAGTTCCGTAATGAGGCTGAGACTTTCAGAAACCAAGCGGAGGGCTTTAAGAACGAGTCCAGTACCAACGCTACGAACACAAAGCAGTGGCGCGATGAGACCAAGGGTTTCCGAGACGAAGCCGAGCGGTTCAAGAATACGGCTGGTCaa                                                                                                                    >  1:285122/1‑301 (MQ=255)`  
                                                                                                                                                                                         `cTATCGGTGTCAATAACGATGGTCACTTGGATGCTCGTGGTCGTCGAATTGTGAACCTAGCGAACGCCGTGGATGACCGCGATGCTGTTCCGTTTGGTCAACTAAAGACCATGGACCAGAACTCATGGCAAGCACGTAATGAAGCCTTACAGTTCCGTAATGAGGCTGAGACTTTCAGAAACCAAGCGGAGGGCTTTAAGAACGAGTCCAGTACCAACGCTACGAACACAACGCAGTGGCGCGATGAGACCAAGGGTTTCCGCGACGAAGCCGAGCGGTTCAAGaactgtctcttatacac                                                                                                                  >  1:238472/1‑286 (MQ=255)`  
                                                                                                                                                                                          `tcggagatgtgtataagagacagaCTTGGATGCTCGTGGTCGTCGAATTGTGAACCTAGCGAACGCCGTGGATGACCGCGATGCTGTTCCGTTTGGTCAACTAAAGACCATGGACCAGAACTCATGGCAAGCACGTAATGAAGCCTTACAGTTCCGTAATGAGGCTGAGACTTTCAGAAACCAAGCGGAGGGCTTTAAGAACGAGTCCAGTACCAACGCTACGAACACAAAGCAGTGGCGCGATGAGACCAAGGGTTTCCGAGACGAAGCCGAGCGGTTCAAGAATACGGCTGGTCAATAc                                                                                                                 <  1:383157/278‑1 (MQ=255)`  
                                                                                                                                                                                           `aTCGGTGTCAATAACGATGGTCACTTGGATGCTCGTGGTCGTCGAATTGTGAACCTAGCGAACGCCGTGGATGACCGCGATGCTGTTCCGTTTGGTCAACTAAAGACCATGGACCAGAACTCATGGCAAGCACGTAATGAAGCCTTACAGTTCCGTAATGAGGCTGAGACTTTCAGAAACCAAGCGGAGGGCTTTAAGAACGAGTCCAGTACCAACGCTACGAACACAAAGCAGTGGCGCGATGAGACCAAGGGTTTCCGAGACGAAGCCAAGCGGTTCAAGGATACGGCTGGTCAATACg                                                                                                                >  1:277522/1‑301 (MQ=255)`  
                                                                                                                                                                                           `aTCGGTGTCAATAACGATGGTCACTTGGATGCTCGTGGTCGTCGAATTGTGAACCTAGCGAACGCCGTGGATGACCGCGATGCTGTTCCGTTTGGTCAACTAAAGACCATGGACCAGAACTCATGGCAAGCACGTAATGAAGCCTTACAGTTCCGTAATGAGGCTGAGACTTTCAGAAACCAAGCGGAGGGCTTTAAGAACGAGTCCAGTACCAACGCTACGAACACAAAGCAGTGGCGCGATGAGACCAAGGGTTTCCGAGACGAAGCCAAGCGGTTCAAGAATACGGcctgtctcttat                                                                                                                >  1:419568/1‑290 (MQ=255)`  
                                                                                                                                                                                                  `tCAATAACGATGGTCACTTGGATGCTCGTGGTCGTCGAATTGTGAACCTAGCGAACGCCGTGGATGACCGCGATGCTGTTCCGTTTGGTCAACTAAAGACCATGGACCAGAACTCATGGCAAGCACGTAATGAAGCCTTACAGTTCCGTAATGAGGCTGAGACTTTCAGAAACCAAGCGGAGGGCTTTAAGAACGAGTCCAGTACCAACGCTACGAACACAAAGCAGTGTCGCGATGAGACCAAGGGTTTCCGAGACGAAGCCGAGCGGTTCAAGAATACGGCTGGTCAATACGCTACATc                                                                                                         <  1:537675/301‑1 (MQ=255)`  
                                                                                                                                                                                                     `agagacagggTCACTTGGATGCTCGTGGTCGTCGAATTGTGAACCTAGCGAACGCCGTGGATGACCGCGATGCTGTTCCGTTTGGTCAACTAAAGACCATGGACCAGAACTCATGGCAAGCACGTAATGAAGCCTTACAGTTCCGTAATGAGGCTGAGACTTTCAGAAACCAAGCGGAGGGCTTTAAGAACGAGTCCAGTACCAACGCTACGAACACAAAGCAGTGGCGCGATGAGACCAAGGGTTTCCGAGACGAAGCCGAGCGGTTCAAGAATACGGCTGGTCAATACGCTACATctgc                                                                                                      <  1:256816/293‑1 (MQ=255)`  
                                                                                                                                                                                                     `aTAACGATGGTCACTTGGATGCTCGTGGTCGTCGAATTGTGAACCTAGCGAACGCCGTGGATGACCGCGATGCTGTTCCGTTTGGTCAACTAAAGACCATGGACCAGAACTCATGGCAAGCACGTAATGAAGCCTTACAGTTCCGTAATGAGGCTGAGACTTTCAGAAACCAAGCGGAGGGCTTTAAGAACGAGTCCAGTACCAACGCTACGAACACAAAGCAGTGGCGCGATGAGACCAAGGGTTTCCGAGACGAAGCCAAGCGGTTCAAGAATACGGCTGGTCAATACGCTACATctgc                                                                                                      <  1:552223/301‑1 (MQ=255)`  
                                                                                                                                                                                                            `tGGTCACTTGGATGCTCGTGGTCGTCGAATTGTGAACCTAGCGAACGCCGTGGATGACCGCGATGCTGTTCCGTTTGGTCAACTAAAGACCATGGACCAGAACTCATGGCAAGCACGTAATGAAGCCTTACAGTTCCGTAATGAGGCTGAGACTTTCAGAAACCAAGCGGAGGGCTTTAAGAACGAGTCCAGTACCAACGCTACGAACACAAAGCAGTGGCGCGATGAGACCAAGGGTTTCCGAGACGAAGCCGAGCGGTTCAAGAATACGGCTGGTCAATACGCTACCTCTGCTGGGAAc                                                                                               >  1:141875/1‑301 (MQ=255)`  
                                                                                                                                                                                                              `gtaaCTTGGATGCTCGTGGTCGTCGAATTGTGAACCTAGCGAACGCCGTGGATGACCGCGATGCTGTTCCGTTTGGTCAACTAAAGACCATGGACCAGAACTCATGGCAAGCACGTAATGAAGCCTTACAGTTCCGTAATGAGGCTGAGACTTTCAGAACCCAAGCGGCGGGCTTTAAGAACGAGTCCAGTACCAACGCTACGAACACAAAGCCGTGTCGCGATGAGACCAAGGGTTTCCGAGACGAAGCCGAGCGGTTCCAGAATACGGCTGGTCACTACGCTACATCTCCTGTGAActc                                                                                             >  1:250840/4‑301 (MQ=255)`  
                                                                                                                                                                                                                    `tGGATGCTCGTGGTCGTCGAATTGTGAACCTAGCGAACGCCGTGGATGACCGCGATGCTGTTCCGTTTGGTCAACTAAAGACCATGGACCAGAACTCATGGCAAGCACGTAATGAAGCCTTACAGTTCCGTAATGAGGCTGAGACTTTCAGAAACCAAGCGGAGGGCTTTAAGAACGAGTCCAGTACCAACGCTACGAACACAAAGCAGTGGCGCGATGAGACCAAGGGTTTCCGAGACGAAGCCAAGCGGTTCAAGAATACGGCTGGTCAATACGCTACATctctgtctcttatacacat                                                                                       >  1:128420/1‑284 (MQ=255)`  
                                                                                                                                                                                                                             `ataagagacagaTTGTGAACCTAGCGAACGCCGTGGATGACCGCGATGCTGTTCCGTTTGGTCAACTAAAGACCATGGACCAGAACTCATGGCAAGCACGTAATGAAGCCTTACAGTTCCGTAATGAGGCTGAGACTTTCAGAAACCAAGCGGAGGGCTTTAAGAACGAGTCCAGTACCAACGCTACGAACACAAAGCAGTGGCGCGATGAGACCAAGGGTTTCCGAGACGAAGCCAAGCGGTTCAAGAATACGGCTGGTCAATACGCTACATCTGCTGGGAACTCTGCTTCCGCTGCGCa                                                                              <  1:183017/290‑1 (MQ=255)`  
                                                                                                                                                                                                                                   `gtcgAATTGTGAACCTAGCGAACGCCGTGGATGACCGCGATGCTGTTCCGTTTGGTCAACTAAAGACCATGGACCAGAACTCATGGCAAGCACGTAATGAAGCCTTACAGTTCCGTAATGAGGCTGAGACTTTCAGAAACCAAGCGGAGGGCTTTAAGAACGAGTCCAGTACCAACGCTACGAACACAAAGCAGTGGCGCGATGAGACCAAGGGTTTCCGAGACGAAGCCGAGCGGTTCAAGAATACGGCTGGTCAATACGCTACATCTGCTTGGTACTCTACTTCCGCTGCGCATCACTc                                                                        >  1:476800/1‑301 (MQ=255)`  
                                                                                                                                                                                                                                         `ttGTGAACTGAGCGAACGCCGTGGAGGACCGGGATGCTGTTCAGTTTGGTCAACTAAAGACCATGGACCAGAACTCATGGCAAGCACGTAATGAAGCCTGACAGTTCCGTAATGAGGCGGAGACTGTCAGAAACCAAGCGGAGGGCTTTAAGAACGAGTCCAGTACCAACGCTACGAACACAAAGCAGTGGCGCGATGAGACCAAGGGTTTCCGAGACGAAGCCAAGCGGTTCAAGAATACGGCTGGTCAATACGCTACATCTGCTGGGAACTCTGCTTCCGCTGCGCATCAATCTGAGGt                                                                  <  1:613514/301‑1 (MQ=255)`  
                                                                                                                                                                                                                                             `ggacagaGCGAACGCCGTGGATGACCGCTATGCTAGTCCGTTTGGTAAACTAAAGACCATGGAACAGAACTCATGGCAAGCACGTAATGAAGCCTTACAGTTCCGTAATGAGGCTGAGACTTTCAGAAACCAAGCGGAGGGCTTTAAGAACGAGACTAGTACCAACGCTACGAACACAAAGCAGTGGCGCGATGAGACCAAGGGTTTCCGAGACGAAGCCGAGCGGTTCAAGAATACGGCTGGTAAATACGCTACATCTGCTGGGAACTCTGCTTCCGCTGCGCATCAATCTGAGGTAAAc                                                              <  1:769430/295‑1 (MQ=255)`  
                                                                                                                                                                                                                                                   `aGCGAACGCCGTGGATGACCGCGATGCTGTTCCGTTTGGTCAACTAAAGACCATGGACCAGAACTCATGGCAAGCACGTAATGAAGCCTTACAGTTCCGTAATGAGGCTGAGACTTTCAGAAACCAAGCGGAGGGCTTTAAGAACGAGTCCAGTACCAACGCTACGAACACAAAGCAGTGTCGCGATGAGACCAAGGGTTTCCGAGACGAAGCCAAGCGGTTCAAGAATACGGCTGGTCAATACGCTACATCTGCTGGGAACTCTGCTTCCGCTGCGCATCAATCTGAGGTAAACGCTgag                                                        >  1:207218/1‑301 (MQ=255)`  
                                                                                                                                                                                                                                                                `gATGACCGCGATGCTGTTCCGTTTGGTCAACTAAAGACCATGGACCAGAACTCATGGCAAGCACGTAATGAAGCCTTACAGTTCCGTAATGAGGCTGAGACTTTCAGAAACCAAGCGGAGGGATTTAAGAACGAGTCCAGTACCAACGCTACGAACACAAAGCAGTGGCGCGATGAGACCAAGGGTTTCCGAGACGAAGCCAAGCGGTTAAAGAATACGGCTGGTAAATACGCTACATCTGCTGGGAACTCTGCTTCCGCTGCGCATCAATCTGAGGTAAACGCTGAGAACTCTGCCACAg                                           <  1:322266/301‑1 (MQ=255)`  
                                                                                                                                                                                                                                                                  `tGACCGCGATGCTGTTCCGTTTGGTCAACTAAAGACCATGGACCAGAACTCATGGCAAGCACGTAATGAAGCCTTACAGTTCCGTAATGAGGCTGAGACTTTCAGAAACCAAGCGGAGGGCTTTAAGAACGAGTCCAGTACCAACGCTACGAACACAAAGCAGTGGCGCGATGAGACCAAGGGTTTCCGAGACGAAGCCAAGCGGTTCAAGAATACGGCTGGTCAATACGCTACATCTGCTGGGAACTCTGCTTCCGCTGCGCATCAATCTGAGGTAAACGCTGAGAACTCTGCCAAAGCa                                         <  1:437620/301‑1 (MQ=255)`  
                                                                                                                                                                                                                                                                             `tgtgtataagagacagaCTAAAGACCATGGACCAGAACTCATGGCAAGCACGTAATGAAGCCTTACAGTTCCGTAATGAGGCTGAGACTTTCAGAAACCAAGCGGAGGGCTTTAAGAACGAGTCCAGTACCAACGCTACGAACACAAAGCAGTGGCGCGATGAGACCAAGGGTTTCCGAGACGAAGCCGAGCGGTTCAAGAATACGGCTGGTCAATACGCTACATCTGCTGGGAACTCTGCTTCCGCTGCGCATCAATCTGAGGTAAACGCTGAGAACTCTGCCACAGCATCCGCTAActa                              <  1:604892/285‑2 (MQ=255)`  
                                                                                                                                                                                                                                                                             `cTGTTCCGTTTGGTCAACTAAAGACCATGGACCAGAACTCATGGCAAGCACGTAATGAAGCCTTACAGTTCCGTAATGAGGCTGAGACTTTCAGAAACCAAGCGGAGGGCTTTAAGAACGAGTCCAGTACCAACGCTACGAACACAAAGCAGTGGCGCGATGAGACCAAGGGTTTCCGAGACGAAGCCAAGCGGTTCAAGAATACGGCTGGTCAATACGCTACATCTGCTGGGAACTCTGCTTCCGCTGCGCATCAATCTGAGGTAAACGCTGAGAACTCTGCCACAGCAGCCGCTAActc                              <  1:190600/301‑1 (MQ=255)`  
                                                                                                                                                                                                                                                                             `cTGTTCCGTTTGGTCAACTAAAGACCATGGACCAGAACTCATGGCAAGCACGTAATGAAGCCTTACAGTTCCGTAATGAGGCTGAGACTTTCAGAAACCAAGCGGAGGGCTTTAAGAACGAGTCCAGTACCAACGCTACGAACACAAAGCAGTGGCGCGATGAGACCAAGGGTTTCCGAGACGAAGCCAAGCGGTTCAAGAATACGGCTGGTCAATACGCTACATCTGCTGGGAACTCTGCTTCCGCTGCGCATCAATCTGAGGTAAACGCTGAGAACTCTGCCACAGCAGCCGCTAActc                              <  1:629246/301‑1 (MQ=255)`  
                                                                                                                                                                                                                                                                                  `ccGTTTGGTCAACTAAAGACCATGGACCAGAACTCATGGCAAGCACGTAATGAAGCCTTACAGTTCCGTAATGAGGCTGAGACTTCCAGAAACCAAGCGGAGGGCTTTAAGAACGAGTCCAGTACCAACGCTACGAACACAAAGCAGTGGCGCGATGAGACCAAGGGTTTCCGAGACGAAGCCAAGCGGTTCAAGAATACGGCTGGTCAATACGCTACATCTGCTGGGAACTCTGCTTCCGCTGCGCATCAATCTGAGGTAAACGCTGAGAACTATGCCACAGCATCCGCTAACTTTGCTc                         >  1:306218/1‑301 (MQ=255)`  
                                                                                                                                                                                                                                                                                             `aCTAAAGACCATGGACCAGAACTCATGGCAAGCACGTAATGAAGCCTTACAGTTCCGTAATGAGGCTGAGACTTTCAGAAACCAAGCGGAGGGCTTTAAGAACGAGTCCAGTACCAACGCTACGAACACAAAGCAGTGGCGCGATGAGACCAAGGGTTTCCGAGACGAAGCCGAGCGGTTCAAGAATACGGCTGGTCAATACGCTACATCTGCTGGGAACTCTGCTTCCGCTGCGCATCAATCTGAGGTAAACGCTGAGAACTCTGCCACAGCATCCGCTAACTTTGCTcctgtctcttat              >  1:107349/1‑290 (MQ=255)`  
                                                                                                                                                                                                                                                                                             `aCTAAAGACCATGGACCAGAACTCATGGCAAGCACGTAATGAAGCCTTACAGTTCCGTAATGAGGCTGAGACTTTCAGAAACCAAGCGGAGGGCTTTAAGAACGAGTCCAGTACCAACGCTACGAACACAAAGCAGTGGCGCGATGAGACCAAGGGTTTCCGAGACGAAGCCAAGCGGTTCAAGAATACGGCTGGTCAATACGCTACATCTGCTGGGAACTCTGCTTCCGCTGCGCATCAATCTGAGGTAAACGCTGAGAACTCTGCCACAGCATCCGCTAACTATGCTCATTTGGCAGaa              >  1:160143/1‑301 (MQ=255)`  
                                                                                                                                                                                                                                                                                                    `aCCATGGACCAGAACTCATGGCAAGCACGTAATGAAGCCTTACAGTTCCGTAATGAGGCTGAGACTTTCAGAAACCAAGCGGAGGGCTTTAAGAACGAGTCCAGTACCAACGCTACGAACACAAAGCAGTGGCGCGATGAGACCAAGGGTTTCCGAGACGAAGCCAAGCGGTTCAAGGATACGGCTGGTCAATACGCTACATCTGCTGGGAACTCTGCTTCCGCTGCGCATCAATCTGAGGTAAACGCTGAGAACTCTGCCACAGCAGCCGCTAACTCTGCTCATTTGGCAGAACagcaag       >  1:445117/1‑301 (MQ=255)`  

                                                                                                                                                                                                                                                                                                          `|`                                                                                                                                                                                                                                                                                                             
`CTCTCTGACAAAGGCTTGGGGTCCAGCCGATGGCTACACGACCATCGAGTTACGTCGAGTAACCTCCACTACCGACCGATTGGTTGACTTTACGGATGGTTCAATCCTCCGCGCGTATGACCTTAACGTCGCTCAGATTCAAACGATGCACGTAGCGGAAGAGGCCCGTGACCTCACTACGGATACTATCGGTGTCAATAACGATGGTCACTTGGATGCTCGTGGTCGTCGAATTGTGAACCTAGCGAACGCCGTGGATGACCGCGATGCTGTTCCGTTTGGTCAACTAAAGACCATGAACCAGAACTCATGGCAAGCACGTAATGAAGCCTTACAGTTCCGTAATGAGGCTGAGACTTTCAGAAACCAAGCGGAGGGCTTTAAGAACGAGTCCAGTACCAACGCTACGAACACAAAGCAGTGGCGCGATGAGACCAAGGGTTTCCGAGACGAAGCCAAGCGGTTCAAGAATACGGCTGGTCAATACGCTACATCTGCTGGGAACTCTGCTTCCGCTGCGCATCAATCTGAGGTAAACGCTGAGAACTCTGCCACAGCATCCGCTAACTCTGCTCATTTGGCAGAACAGCAAGCAGAC  >  NC_001604.1/34797‑35394`

  

Alignment Legend

Aligned base mismatch/match (shaded by quality score): `ATCG/ATCG` `< 3 ≤` `ATCG/ATCG` `< 11 ≤` `ATCG/ATCG` `< 25 ≤` `ATCG/ATCG` `< 36 ≤` `ATCG/ATCG` `< 38 ≤` `ATCG/ATCG`

Unaligned base: `atcg`    Masked matching base: `atcg`    Alignment gap: `‑`    Deleted base: `‑`
